# Antidepressants during pregnancy and autism in offspring: population based cohort study

**DOI:** 10.1136/bmj.j2811

**Published:** 2017-07-19

**Authors:** Dheeraj Rai, Brian K Lee, Christina Dalman, Craig Newschaffer, Glyn Lewis, Cecilia Magnusson

**Affiliations:** 1 Centre for Academic Mental Health, School of Social and Community Medicine, University of Bristol, Bristol, UK; 2 Avon and Wiltshire Partnership NHS Mental Health Trust, Bristol, UK; 3 Department of Public Health Sciences, Karolinska Institutet, Stockholm, Sweden; 4 NIHR Biomedical Research Centre, University of Bristol, Bristol, UK; 5 Drexel University School of Public Health, Philadelphia, PA, USA; 6 AJ Drexel Autism Institute, Philadelphia, PA, USA; 7 Centre for Epidemiology and Community Medicine, Stockholm Health Care Services, Stockholm, Sweden; 8 Division of Psychiatry, University College London, London, UK

## Abstract

**Objectives** To study the association between maternal use of antidepressants during pregnancy and autism spectrum disorder (ASD) in offspring.

**Design** Observational prospective cohort study with regression methods, propensity score matching, sibling controls, and negative control comparison.

**Setting** Stockholm County, Sweden.

**Participants** 254 610 individuals aged 4-17, including 5378 with autism, living in Stockholm County in 2001-11 who were born to mothers who did not take antidepressants and did not have any psychiatric disorder, mothers who took antidepressants during pregnancy, or mothers with psychiatric disorders who did not take antidepressants during pregnancy. Maternal antidepressant use was recorded during first antenatal interview or determined from prescription records.

**Main outcome measure** Offspring diagnosis of autism spectrum disorder, with and without intellectual disability.

**Results** Of the 3342 children exposed to antidepressants during pregnancy, 4.1% (n=136) had a diagnosis of autism compared with a 2.9% prevalence (n=353) in 12 325 children not exposed to antidepressants whose mothers had a history of a psychiatric disorder (adjusted odds ratio 1.45, 95% confidence interval 1.13 to 1.85). Propensity score analysis led to similar results. The results of a sibling control analysis were in the same direction, although with wider confidence intervals. In a negative control comparison, there was no evidence of any increased risk of autism in children whose fathers were prescribed antidepressants during the mothers’ pregnancy (1.13, 0.68 to 1.88). In all analyses, the risk increase concerned only autism without intellectual disability.

**Conclusions** The association between antidepressant use during pregnancy and autism, particularly autism without intellectual disability, might not solely be a byproduct of confounding. Study of the potential underlying biological mechanisms could help the understanding of modifiable mechanisms in the aetiology of autism. Importantly, the absolute risk of autism was small, and, hypothetically, if no pregnant women took antidepressants, the number of cases that could potentially be prevented would be small.

## Introduction

Depression is common in women of childbearing age, and in Europe 3-8% of pregnant women are prescribed antidepressants during pregnancy.^1^ The fetal safety of antidepressant exposure during pregnancy has generated much debate after recent concerns of a possible association with autism in exposed offspring. In the past five years, several epidemiological studies^2^ ^3^ ^4^ ^5^ ^6^ ^7^ ^8^ ^9^ ^10^ ^11^ have assessed the relation between antidepressant use during pregnancy and autism in offspring, but robust conclusions have been elusive.^12^ ^13^ Although most studies found evidence of unadjusted associations, conclusions differed because of concerns about “confounding by indication.” This was because depression or other psychiatric indications for antidepressant use could be associated with autism through genetic or non-genetic pathways, and thus the possibility of the observed associations representing the risk of autism from the underlying indication for prescription could not be ruled out.

All antidepressants cross the placental barrier and are available to the developing fetus.^14^ Most of the commonly used antidepressants such as selective serotonin reuptake inhibitors (SSRIs) increase the availability of serotonin in the synaptic cleft. The serotonergic system emerges early in embryogenesis and is critical for neurodevelopment.^15^ In utero exposure to serotonergic antidepressants in animal models have reported associations with autism-like behaviours in the offspring.^16^ It is therefore biologically plausible that similar effects could be seen in humans. Disentangling a potential causal association of antidepressants on the risk of autism from that observed from confounding by indication is crucial to reduce clinical uncertainty and help women make informed decisions regarding the risks and benefits of antidepressant use during pregnancy.

In the absence of randomised controlled trials,^7^ however, observational studies are the only available source of making risk:benefit decisions in relation to antidepressant use during pregnancy. It is well known that such studies are prone to confounding bias, which can persist even after adjustment for multiple confounders.^17^ ^18^ ^19^ Several approaches—such as propensity score matching, negative controls, and sibling control designs—have been suggested as strategies that could strengthen causal inference in observational studies^18^ but remain largely unused in investigations of this issue. To help to improve the understanding of the association between antidepressant use during pregnancy and autism in offspring, we applied a range of such causal analytical methods on data from a large total population cohort in Stockholm County. We hypothesised that if the association between antidepressant use during pregnancy and autism was likely to be causal, the results would be consistent across a range of analytical methods with different strengths and limitations and underlying assumptions.

## Methods

### Stockholm youth cohort

We used data from the Stockholm youth cohort, an intergenerational record linkage study comprising all individuals aged 0-17 living in Stockholm County in 2001-11 (n=735 096). It contains prospectively recorded data on the cohort members and their first degree relatives collected by record linkage with a range of national and regional healthcare, social, and administrative registries.^20^ ^21^ The key for record linkage is the unique national identity numbers assigned to all Swedish residents. Figure 1 shows the derivation of the sample for our main analysis. We excluded cohort members born before 1996 as medication data were reliably collected only after this date. We also excluded individuals not linked to the medical birth register (such as those born abroad), those who could not be linked to their biological mothers, adopted children, and those living in Stockholm County for less than four years. The residence requirement also allowed us to exclude children aged under 4 in whom a diagnosis of autism might be less reliable.

**Figure f1:**
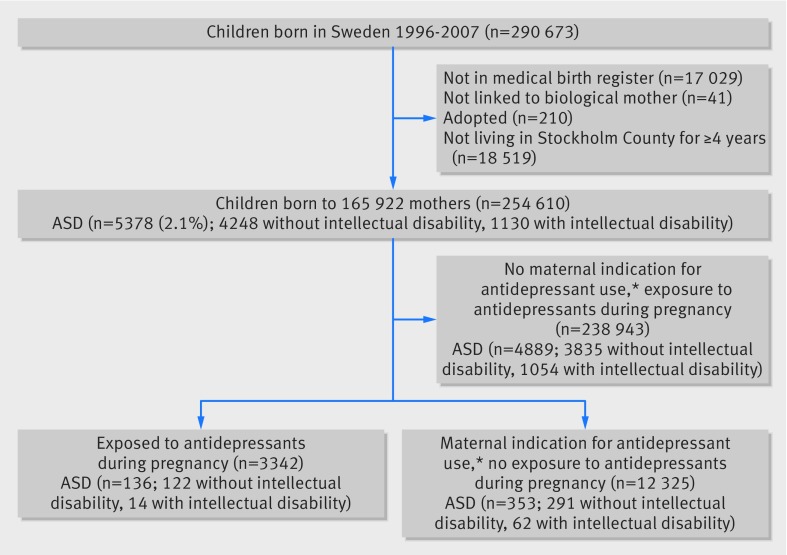
**Fig 1** Derivation of sample from Stockholm youth cohort used in main analysis of maternal use of antidepressants during pregnancy and autism in offspring. *Maternal indications for antidepressant use were anxiety disorders (F40-41), bipolar disorder (F30-31), depression/mood disorder (F32-39), non-affective psychoses (F20-29), obsessive compulsive disorders (F42), stress related disorders (F43), and other neurotic disorders (F44-48)

### Patient involvement

As mandated by the ethical permission, no attempts were made to contact any cohort members for any aspect of this record linkage study. As such, cohort members were not involved in setting the research question or the outcome measures, nor were they involved in developing plans for the design or implementation of the study. No patients were asked to advise on interpretation or writing up of results. There are no plans to directly disseminate the results of the research to cohort members, but dissemination to the general public and relevant patient groups will be undertaken by using presentations and social media.

### Medication use during pregnancy (exposure)

We derived information on maternal use of antidepressants in pregnancy from the medical birth register (since 1997) and supplemented it with the prescribed drug register (available from July 2005).^22^ ^23^ The medical birth register contains information on current medications being taken as reported by pregnant women at their antenatal interview, at a median of 10 weeks’ gestation. The data are semi-automatically coded with World Health Organization anatomical therapeutic classification (ATC) codes. The medical birth register also contains free text data, which we processed using a computerised search for generic drug names and Swedish and international brand names of antidepressants using fuzzy pattern matching to account for unknown abbreviations, non-standard terms, and misspellings.^24^ The prescribed drug register contains data on drugs prescribed and dispensed in ambulatory care to the entire Swedish population, and the medications are coded with ATC codes. In the prescribed drug register, we defined exposure to medication during pregnancy as a prescription up to 30 days before the start of the pregnancy (as estimated by the last menstrual period or evidence from ultrasonography) until the birth date of the child. We considered exposure to antidepressants if there was a record of these in either the medical birth register or the prescribed drug register. The validity of these two data sources have been reported previously.^22^ ^23^ We cross validated these two data sources in our sample as data on antidepressant use were available in both registers for cohorts born in 2006 and 2007. Among these, 280 women out of 318 with a report of antidepressant use in the medical birth register were also recorded as having prescriptions for an antidepressant during pregnancy in the prescribed drug register (88.1%).

We coded the medications as any antidepressant (ATC code N06A) and also divided them into selective serotonin reuptake inhibitors (SSRI, ATC N06AB) and all other antidepressants. We also categorised antidepressants based on their affinities for the serotonin transporter into high versus medium or low affinity.^2^

### Ascertainment of autism with and without intellectual disability (outcome)

We have described the multisource ascertainment of autism in previous publications.^7^ ^20^ ^21^ In short, nearly all diagnoses of autism in Sweden are provided through its free and universally accessible system of health and care. We collected diagnostic information from the relevant registers reflecting the pathways to diagnosis. These sources included diagnoses recorded in the national patient register, the Stockholm child and adolescent mental health register, and the habilitation registers (ICD-9 (299), ICD-10 (F84), or DSM-IV (299) codes). We identified co-occurring intellectual disability using ICD 9 (317-319), ICD-10 (F70-79), and DSM-IV (317-319) in the child or adult mental health registers or the national patient register. We have previously carried out two validation procedures— a case note validation study and a cross validation study of diagnosis of autism spectrum disorder with a national twin study—and found a high validity of the diagnoses recorded in the registers.^20^

### Other variables

#### Depression and other psychiatric disorders in parents

We used ICD-9 and 10 diagnoses recorded in the national patient register, which covers inpatient (with complete national coverage since 1973) and outpatient specialist care (since 2001) to ascertain depression and other maternal and paternal diagnoses of other psychiatric indications for antidepressants (anxiety disorder, bipolar disorder, non-affective psychoses, obsessive-compulsive disorder, other stress related and neurotic disorders) any time before the birth of the child as described elsewhere.^25^ We supplemented these with diagnoses recorded from the Stockholm adult psychiatric care register, which comprises all publicly financed psychiatric care in Stockholm County (constituting 85% of all such care) since 1997.^26^

We used prospectively collected data on maternal and paternal age at birth of child (continuous variables used as restricted cubic splines), fifths of family income adjusted for year of ascertainment and family size, highest education of either parent (≤9 years, 10-12 years, ≥13 years), maternal country of birth (Sweden, Europe, other), parity (0, 1, 2, or more previous births) as potential confounders. We also used several other variables to construct the propensity score as described below. These included maternal smoking and body mass index (BMI) recorded at first antenatal visit, number of diagnoses of maternal depression before birth, type of depression care (inpatient or outpatient), and a large range of maternal neurological and psychiatric conditions diagnosed before birth (see appendix).

### Main analysis

We used R-3.1.3 (R Foundation for Statistical Computing) for analysis. After descriptive analyses, we used the following analytic strategies to assess the risk of offspring autism in mothers with antidepressant use during pregnancy.

#### Analysis 1: risk estimates of autism in children of antidepressant users during pregnancy compared with those with psychiatric disorders but no antidepressant use

In our first analysis, we used logistic regression to derive odds ratios and their 95% confidence intervals as estimates of relative risks for autism in children of mothers who used antidepressants during pregnancy compared with those with a psychiatric disorder who did not use antidepressants. We used this stringent comparison group to better account for confounding by indication and because women without any psychiatric disorder would be ineligible in hypothesised randomised controlled trials of this issue. We adjusted the model for birth year to control for period effects in medication use and ascertainment of autism.^21^ We then adjusted for the presence or absence of specific individual maternal psychiatric disorders (depression, anxiety disorder, bipolar disorder, non-affective psychotic disorders, obsessive-compulsive disorder, adjustment disorders, post-traumatic stress disorders, and other neurotic disorders) that are indications for antidepressant use (model 2) and additionally for child sex, parental ages, birth order, maternal education, family income, and maternal country of birth (model 3). We calculated population attributable fractions (PAF) using the fully adjusted model, estimating the proportion of autism cases that would be prevented if no women with psychiatric disorders were prescribed antidepressants, assuming a causal association and no residual confounding. In a supplementary analysis, we restricted the above treatment group to mothers with antidepressant use who had a recorded psychiatric diagnosis. We also described the associations by the most commonly used individual antidepressants and grouped antidepressants as SSRI and non-SSRIs, and by their serotonin transporter receptor (SERT) affinity into high or low/medium affinity.

#### Analysis 2: propensity score matched analysis

In our second analysis, we calculated propensity score matched estimates for the above associations. Propensity score matching helps to minimise the potential for confounding from observed variables by comparing exposed and unexposed individuals with similar characteristics.^27^ We estimated propensity scores for antidepressant use during pregnancy using a boosted classification and regression tree model,^28^ from the above covariates and additional variables regarding maternal psychiatric disorders including indicators of severity such as the type of psychiatric care and number of previous care episodes for depression, and the use of other medications (see appendix for complete details). We matched children of women who did and did not use antidepressants using the propensity score using a maximum of 4:1 unexposed:exposed nearest neighbour matching with a caliper of 0.20 SD and exact matching on birth year, sex of child, number of depression diagnoses, anxiety disorders, and obsessive compulsive disorder. We estimated odds ratios and 95% confidence interval from cluster robust logistic regression models.

#### Analysis 3: outcome discordant matched sibling sets analysis

In our third analysis, we examined associations between maternal antidepressant use in pregnancy and autism spectrum disorder in matched sets of outcome discordant siblings (that is, sibling sets in which there is at least one affected sibling and one unaffected sibling, see appendix). Siblings share up to half of their genetic makeup and generally share their early postnatal environment. Sibling analysis can be a powerful method to control for unmeasured confounders, such as maternal genetic liability for neuropsychiatric conditions, when the confounders are shared more than the exposure.^29^ We derived odds ratios and confidence intervals using conditional logistic regression models adjusted for sex, parity, and birth year.

#### Analysis 4: comparison with risk estimates for paternal antidepressant use during pregnancy as negative control

In our fourth analysis, we used paternal use of antidepressant as a negative control exposure with the assumption that the fathers would share many of the unmeasured confounders with mothers, and, if a similar heightened risk of autism was observable with paternal and maternal use of antidepressants, the associations would be unlikely to reflect an in utero effect of the medications.^30^ In these analyses, we used data for the individuals born in 2006 and 2007, for whom we had data on antidepressant prescriptions for the mothers and fathers, and estimated the associations of antidepressant prescriptions in fathers during the time of the mother’s pregnancy with the outcomes.

### Sensitivity analysis

In a sensitivity analysis, we estimated how robust the estimates of associations between antidepressant exposure and autism spectrum disorder were to unmeasured confounding in our propensity score matched sample. We assumed a binary confounder *U* increased the risk of autism and was more prevalent in antidepressant users than in non-antidepressant users. Given specified parameters such as the relation of *U* with autism spectrum disorder and the prevalence of *U* in antidepressant users and non-users, we estimated odds ratios and 95% confidence interval corrected for this unmeasured confounder, specified over a range of plausible parameters.^31^

### Missing data

There were few missing data on the key variables in our main analysis (total 1.5% missing data). More data were missing in some variables used in the generation of propensity scores, but the boosted classification and regression tree model is able to incorporate missing values in the prediction (see appendix).

## Results

Table 1 shows the descriptive statistics of the cohort in relation to exposure to antidepressants during pregnancy or mental disorders. Of the 3342 children exposed to antidepressants during pregnancy, 136 (4.1%) had a diagnosis of autism. The comparison group included 12 325 children of mothers with a psychiatric disorder who did no use antidepressants during pregnancy, of whom 353 (2.9%) had a diagnosis of autism. Of the 238 943 cohort children for whom there was no record of maternal history of psychiatric disorder or antidepressant use during pregnancy, 4889 had autism (2.1%). The sample sizes for all of the other analyses are provided in the appendix.

**Table 1 tbl1:** Selected characteristics of Stockholm youth cohort. Figures are numbers (percentage) unless stated otherwise

	Exposed to antidepressants during pregnancy (n=3342)	Maternal psychiatric disorder and unexposed to antidepressants (n=12 325)	No maternal psychiatric disorder and unexposed to antidepressants (n=238 943)
Autism spectrum disorder (ASD)	136 (4.1)	353 (2.9)	4889 (2.1)
ASD without intellectual disability	122 (3.7)	291 (2.4)	3835 (1.6)
ASD with intellectual disability	14 (0.4)	62 (0.5)	1054 (0.4)
Mean (SD) maternal age (years)	31.7 (5.2)	31.5 (5.4)	30.7 (5.0)
Mean (SD) paternal age (years)	34.0 (6.4)	34.0 (6.6)	33.7 (6.1)
Male child	1727 (51.7)	6446 (52.3)	122 354 (51.2)
Parity:			
1	1673 (50.1)	5203 (42.2)	108 192 (45.3)
2	951 (28.4)	4248 (34.5)	87 765 (36.7)
≥3	718 (21.5)	2874 (23.3)	42 986 (18.0)
Maternal education >12 years	1382 (41.3)	4897 (39.7)	110 017 (46.0)
Family income in highest fifth	904 (27.1)	3306 (26.8)	83 190 (34.8)
Mother born in Sweden	2,713 (81.2)	9176 (74.5)	177 395 (74.2)
Mother smoked	512 (15.3)	1434 (11.6)	16 996 (7.1)
Maternal BMI at 1st antenatal visit:			
Normal (18.5-<25)	1741 (52.1)	6035 (49.0)	129 597 (54.2)
Underweight (<18.5)	81 (2.4)	316 (2.6)	5590 (2.3)
Overweight (25-<30)	619 (18.5)	2272 (18.4)	40 212 (16.8)
Obese (≥30)	328 (9.8)	981 (8.0)	13 945 (5.8)
Missing	573 (17.2)	2721 (22.1)	49 599 (20.8)
Maternal lifetime psychiatric diagnoses before birth:			
Depression	1378 (41.3)	5800 (47.1)	0
Anxiety disorder	685 (20.5)	2594 (21.1)	0
Bipolar disorder	70 (2.1)	402 (3.3)	0
Non-affective psychoses	46 (1.4)	645 (5.2)	0
Obsessive-compulsive disorder	56 (1.7)	139 (1.1)	0
Stress related disorders	389 (11.7)	4261 (34.6)	0
Other neurotic disorders	89 (2.7)	1202 (9.8)	0
No of diagnoses of maternal depression before birth:			
0	1964 (58.8)	6525 (52.9)	238 943 (100)
1	517 (15.5)	3287 (26.7)	0
2	240 (7.2)	1128 (9.2)	0
3	172 (5.2)	508 (4.1)	0
≥4	449 (13.4)	877 (7.1)	0
No of diagnoses of maternal depression before birth by treatment:			
Specialist care:			
0	2337 (69.9)	8018 (65.1)	238 943 (100)
1	388 (11.6)	2493 (20.2)	0
2	199 (6.0)	952 (7.7)	0
3	418 (12.5)	862 (7.0)	0
Primary care:			
0	3059 (91.5)	11 766 (95.5)	238 943 (100)
1	129 (3.9)	286 (2.3)	0
≥2	154 (4.6)	273 (2.2)	0
Inpatient diagnosis:			
0	2,982 (89.2)	10 938 (88.8)	238 943 (100)
1	229 (6.9)	969 (7.9)	0
2	70 (2.1)	233 (1.9)	0
≥3	61 (1.8)	184 (1.5)	0
Other:			
0	3201 (95.8)	12 042 (97.7)	238 943 (100)
1	105 (3.1)	232 (1.9)	0
≥2	36 (1.1)	51 (0.4)	0
Medications during pregnancy:			
SSRI antidepressants	2710 (81.1)	—	0
Non-SSRI antidepressants	723 (21.6)	—	0
Antiepileptics	37 (1.1)	73 (0.6)	490 (0.2)
Antipsychotics	106 (3.2)	166 (1.4)	347 (0.2)
Anxiolytics	314 (9.4)	191 (1.6)	337 (0.1)

Exposure to antidepressants during pregnancy was associated with a higher odds of a diagnosis of autism in offspring than exposure to a maternal psychiatric disorder without antidepressants (adjusted odds ratio 1.45, 95% confidence interval 1.13 to 1.85; table 2). This association was observed only for autism without intellectual disability (1.57, 1.21 to 2.04). The results were similar when we restricted the antidepressant exposure group to mothers who had a recorded psychiatric diagnosis (table A in appendix). If we assume an unconfounded causal association, the corresponding population attributable fractions suggested that about 2% of autism cases would be prevented if no pregnant woman with a psychiatric disorder took antidepressants (2.1%, 95% confidence interval −0.7% to 4.7%).

**Table 2 tbl2:** Regression estimated odds ratios and 95% confidence intervals for associations between antidepressant use during pregnancy and autism spectrum disorder (ASD) in children exposed prenatally to antidepressants compared with children exposed to maternal psychiatric disorders but no antidepressants (combined n=15 667) in cluster robust logistic regression models (cluster=birth mother)

Outcome (No of exposed cases)	Model 1^*^	Model 2^†^	Model 3^‡^
ASD (136)	1.47 (1.20 to 1.81)	1.47 (1.16 to 1.87)	1.45 (1.13 to 1.85)
ASD without intellectual disability (122)	1.59 (1.28 to 1.98)	1.62 (1.25 to 2.08)	1.57 (1.21 to 2.04)
ASD with intellectual disability (14)	0.87 (0.49 to 1.57)	0.81 (0.39 to 1.68)	0.72 (0.38 to 1.77)

*Adjusted for birth year.

†Additionally adjusted for maternal psychiatric disorders diagnosed before birth (depression, anxiety disorder, bipolar disorder, non-affective psychotic disorders, obsessive-compulsive disorder, stress related disorders, other neurotic disorders), maternal medications used during pregnancy (antiepileptics, antipsychotics, anxiolytics).

‡Additionally adjusted for sex, maternal age, paternal age, parity, maternal education, family income, maternal birth country.

The propensity score analysis led to similar results to those found with conventional regression models (table 3 and appendix). The numbers were smaller for the sibling control analyses, but results again seemed consistent, though with wider confidence intervals that included 1 (table 3 and appendix). In our negative control analysis, there was no evidence of an increased risk of autism in children whose fathers were prescribed antidepressants during the mothers’ pregnancy (adjusted odds ratio 1.13, 95% confidence interval 0.68 to 1.88), but the association with maternal prescriptions for antidepressants continued to be observed (1.69, 1.06 to 2.72; table 4). In all analyses, the risk estimates were greater for individuals without intellectual disability than those with intellectual disability.

**Table 3 tbl3:** Matching estimated odds ratios and 95% confidence interval for associations between antidepressant use during pregnancy and autism spectrum disorder (ASD)

Outcome	Propensity score matched analysis^*^			Sibling matched analysis^†^	
	Exposed/unexposed	OR (95% CI)		Affected sibling sets/exposed	OR (95% CI)
ASD	1608/4818	1.68 (1.23 to 2.30)		3038/66	1.36 (0.84 to 2.20)
ASD without intellectual disability	1601/4801	1.76 (1.26 to 2.46)		2408/60	1.57 (0.92 to 2.66)
ASD with intellectual disability	1552/4721	1.25 (0.52 to 3.03)		630/6	0.78 (0.24 to 2.54)

*Propensity score matched estimates of associations of antidepressant use during pregnancy and ASD in children exposed prenatally to antidepressants compared with children exposed to maternal psychiatric disorder but no antidepressants.

†Outcome discordant sibling matched estimates for associations of antidepressant use during pregnancy and ASD. Conditional logistic regression models adjusted for sex, parity, and birth year.

**Table 4 tbl4:** Negative control analysis: odds ratios and 95% confidence interval for associations between parental antidepressant use^*^ ascertained through national prescription drug register and autism spectrum disorder (ASD) in Stockholm youth cohort subsample (n=47 629) born 2006-07 in cluster robust logistic regression models (cluster=birth mother).

	OR (95% CI)	
	Unadjusted	Adjusted†
ASD		
Maternal antidepressant use	2.01 (1.34 to 3.01)	1.69 (1.06 to 2.72)
Paternal antidepressant use	1.38 (0.90 to 2.12)	1.13 (0.68 to 1.88)
ASD without intellectual disability		
Maternal antidepressant use	2.27 (1.49 to 3.47)	1.85 (1.11 to 3.09)
Paternal antidepressant use	1.46 (0.92 to 2.32)	1.18 (0.68 to 2.06)
ASD with intellectual disability		
Maternal antidepressant use	0.86 (0.21 to 3.49)	0.83 (0.25 to 2.82)
Paternal antidepressant use	1.03 (0.32 to 3.24)	0.91 (0.26 to 3.27)

*Antidepressant exposure defined as ever/never during pregnancy if prescription dispensed from 30 days before conception until birth.. †Adjusted for birth year, maternal and paternal antidepressant use, maternal and paternal depression before birth, maternal age, sex, paternal age, parity, maternal education, family income fifth, maternal birth country, and other maternal psychiatric disorders before birth (anxiety disorder, bipolar disorder, non-affective psychotic disorders, obsessive-compulsive disorder, stress-related disorders, other neurotic disorders).

In sensitivity analysis, the results seemed to be moderately robust to unmeasured confounding (table B in appendix). Tables C and D in the appendix show the rates and associations of autism in children exposed to the most commonly prescribed individual antidepressants, also grouped into SSRI and non-SSRI antidepressants as well as by their serotonin receptor affinity. Although imprecision because of small numbers is evident, the rates of autism seemed to be higher in children of mothers who used clomipramine and venlafaxine and lowest in users of paroxetine. Similar associations were observed for SSRI and non-SSRI antidepressants in relation to risk. The point estimates for the risk of autism in children of users of low/moderate SERT affinity seemed to be greater than that in children of users of high serotonin transporter affinity antidepressants, though the confidence intervals overlapped.

## Discussion

### Principal findings

In this large Swedish population based study, we carried out several analyses to further investigate the association between antidepressant use during pregnancy and autism in offspring. Our main findings were that children exposed to antidepressants during pregnancy seemed to be at a higher risk of autism, particularly autism without intellectual disability, than children of mothers with psychiatric disorders who were not treated with antidepressants during pregnancy. The findings seemed to be consistent across traditional regression methods, propensity score matching, and a sibling set comparison, and maternal exposure to antidepressants during pregnancy had a strong association with the outcomes whereas no such association was observed with paternal exposure. This points to a potential effect of antidepressant use on the risk of autism beyond any effect caused by confounding by the underlying condition. It is important to note, however, that the absolute risk was small, and 4.1% of children exposed to antidepressants in utero had autism compared with 2.9% of those with a maternal history of psychiatric disorder. We estimated that only about 2% of autism cases in this population would be theoretically prevented, if the association was causal and no women with psychiatric disorders used antidepressants during pregnancy.

### Strengths and limitations

The main strengths of this study were the large sample size and the range of analyses carried out to strengthen causal inference beyond traditional methods. As the study included the total population of Stockholm County and benefited from multisource ascertainment of cases, as opposed to studies that rely on hospital discharge diagnoses, the findings are likely to have high external validity. The possibility of misclassification of exposure cannot be ruled out, but the availability of both self reported information from the medical birth register and dispensation information from the prescribed drug register was an advantage.^22^ ^23^ As the absence of detailed measures of severity of depression during pregnancy was a limitation, we used propensity scores to match and therefore balance exposure groups using a wide range of relevant characteristics. Because of small numbers, we were not able to assess trimester specific or dose response effects.

### Comparison with other studies

This study builds on our previous case-control study,^7^ now enhanced with a larger sample, a more stringent comparison group of mothers with psychiatric disorders, and a range of causal inference methods including propensity score matching, a sibling comparison, and a negative control design to strengthen confidence in the results. Although several large register based studies have been carried out to date, the number of children with autism who were also prenatally exposed to antidepressants has been small, and some studies seemed to have substantially under-ascertained autism. This has led to imprecise estimates, which have been pervasive in all studies on this topic, including those that have concluded that the association is likely to be explained by confounding.^6^ ^8^ Unlike some previous studies, we were unable to study discontinuation of antidepressants before pregnancy as an additional negative control as the prescribed drug register was operational only since 2005 and thus had insufficient numbers. It should be noted that as a large proportion of women discontinue drug treatment during pregnancy,^32^ the cohorts that did have such data^2^ ^10^ ^11^ had more statistical power to find an effect in the discontinuation group, as opposed to effects in those who continued treatment during pregnancy. The upper limits of the estimates of the associations reported in these studies were consistent with the results we report.

### Meaning of the findings

The different analyses we used have different strengths and limitations, but their findings seemed to triangulate, pointing towards an association between maternal antidepressant use in pregnancy and autism in offspring. On the other hand, the increased risk was seen largely for autism without intellectual disability, a phenotype that has been shown to be more heritable,^33^ which could suggest a role for unmeasured genetic confounding. Although the results of our sibling control analyses were consistent with the other approaches we used, the numbers were low, leading to an imprecise result. Larger sibling comparisons in the future could elucidate the role of genetic confounding in this relation. Furthermore, higher point estimates for lower serotonin transporter receptor affinity antidepressants, several of which are prescribed for more severe depression,^2^ could suggest a role of confounding by severity of depression, which is difficult to measure in record linkage studies such as this one. Taken together, it is difficult to conclusively dismiss the possibility that the observed associations are wholly attributable to confounding. We simulated the potential impact of unmeasured confounding, which suggested that such an unmeasured confounder would have to be a strong risk factor for autism, exerting a confounding influence above and beyond the multiple covariates already controlled for in the propensity score matching.

### Clinical implications

So what should families and doctors making decisions about antidepressants during pregnancy make of such results? Firstly, this and other studies clearly suggest that there is an increased background risk of autism in children of women with psychiatric conditions, regardless of antidepressant treatment. Secondly, despite the observed relative risks, over 95% of women who took antidepressants during pregnancy did not have a child with autism. And, finally, if a causal link were robustly established, and if no pregnant women took antidepressants during pregnancy, only 2% of autism cases in this population would be prevented. It is known that pregnant women perceive such risks as greater than they are,^34^ and a balanced discussion in relation to clinical decision making in the light of evolving but yet inconsistent evidence is important. On the other hand, given that this association might not solely be the byproduct of confounding by indication, it is important to continue investigation of possible underlying biological mechanisms that could help us to better understand the aetiology of autism.

**What is already known about this topic**

Several observational studies have reported associations between antidepressant use during pregnancy and autism in offspring
Whether this association is causal or confounded by indication is not clear

**What this study adds**

This large cohort study used various methods to deal with confounding, including traditional multivariable regression, propensity score matching, sibling controls, and a negative control design
The results of all these analyses, which used different assumptions, seemed to be consistent with each other, suggesting that the association between in utero exposure to antidepressants and autism might not be fully explained by confounding
The absolute risks were small so results should not be considered alarming, but the findings could be useful in a further understanding of the aetiology of autism

## Web Extra

Extra material supplied by the author

## Notes

We thank Michael Lundberg, data manager at Karolinska Institutet for help with data extraction.

Contributors: DR and BKL are joint first authors. DR, BKL, and CM had the research idea, and CD, CN, and GL helped with its development. DR wrote the first and subsequent drafts of the paper with important intellectual input from all coauthors. BKL conducted the statistical analysis. All authors had full access to the data, specifically, the statistical reports and tables arising from the data. BKL takes responsibility of the integrity of the data and accuracy of the data analysis. All authors have approved the final version of the manuscript submitted for publication. DR, BKL, and CM are guarantors.

Ethical approval: The study was approved by the research ethics committee at Karolinska Institutet, Stockholm (2013/1118-32). No individual level consent was required, and all data were anonymised before being received by the research team.

Data sharing: No further data available.

Transparency statement: The authors affirm that the manuscript is an honest, accurate, and transparent account of the study being reported; no important aspects of the study have been omitted; and any discrepancies from the study as planned have been explained.

## References

[1] Charlton RA, Jordan S, Pierini A, et al. Selective serotonin reuptake inhibitor prescribing before, during and after pregnancy: a population-based study in six European regions. BJOG 2015;122:1010-20. 10.1111/1471-0528.13143. pmid:25352424. 25352424

[2] Clements CC, Castro VM, Blumenthal SR, et al. Prenatal antidepressant exposure is associated with risk for attention-deficit hyperactivity disorder but not autism spectrum disorder in a large health system. Mol Psychiatry 2015;20:727-34. 10.1038/mp.2014.90 pmid:25155880. 25155880 PMC4427538

[3] Croen LA, Grether JK, Yoshida CK, Odouli R, Hendrick V. Antidepressant use during pregnancy and childhood autism spectrum disorders. Arch Gen Psychiatry 2011;68:1104-12. 10.1001/archgenpsychiatry.2011.73 pmid:21727247. 21727247

[4] Gidaya NB, Lee BK, Burstyn I, Yudell M, Mortensen EL, Newschaffer CJ. In utero exposure to selective serotonin reuptake inhibitors and risk for autism spectrum disorder. J Autism Dev Disord 2014;44:2558-67. 10.1007/s10803-014-2128-4. pmid:24803368. 24803368

[5] Harrington RA, Lee LC, Crum RM, Zimmerman AW, Hertz-Picciotto I. Prenatal SSRI use and offspring with autism spectrum disorder or developmental delay. Pediatrics 2014;133:e1241-8. 10.1542/peds.2013-3406 pmid:24733881. 24733881 PMC4006441

[6] Hviid A, Melbye M, Pasternak B. Use of selective serotonin reuptake inhibitors during pregnancy and risk of autism. N Engl J Med 2013;369:2406-15. 10.1056/NEJMoa1301449. pmid:24350950. 24350950

[7] Rai D, Lee BK, Dalman C, Golding J, Lewis G, Magnusson C. Parental depression, maternal antidepressant use during pregnancy, and risk of autism spectrum disorders: population based case-control study. BMJ 2013;346:f2059 10.1136/bmj.f2059 pmid:23604083. 23604083 PMC3630989

[8] Sørensen MJ, Grønborg TK, Christensen J, et al. Antidepressant exposure in pregnancy and risk of autism spectrum disorders. Clin Epidemiol 2013;5:449-59. 10.2147/CLEP.S53009 pmid:24255601. 24255601 PMC3832387

[9] Boukhris T, Sheehy O, Mottron L, Bérard A. Antidepressant Use During Pregnancy and the Risk of Autism Spectrum Disorder in Children. JAMA Pediatr 2016;170:117-24. 10.1001/jamapediatrics.2015.3356 pmid:26660917. 26660917

[10] Castro VM, Kong SW, Clements CC, et al. Absence of evidence for increase in risk for autism or attention-deficit hyperactivity disorder following antidepressant exposure during pregnancy: a replication study. Transl Psychiatry 2016;6:e708 10.1038/tp.2015.190. pmid:26731445. 26731445 PMC5068870

[11] Malm H, Brown AS, Gissler M, et al. Gestational Exposure to Selective Serotonin Reuptake Inhibitors and Offspring Psychiatric Disorders: A National Register-Based Study. J Am Acad Child Adolesc Psychiatry 2016;55:359-66. 10.1016/j.jaac.2016.02.013. pmid:27126849. 27126849 PMC4851729

[12] Gentile S. Prenatal antidepressant exposure and the risk of autism spectrum disorders in children. Are we looking at the fall of Gods?J Affect Disord 2015;182:132-7. 10.1016/j.jad.2015.04.048. pmid:25985383. 25985383

[13] Kaplan YC, Keskin-Arslan E, Acar S, Sozmen K. Prenatal selective serotonin reuptake inhibitor use and the risk of autism spectrum disorder in children: A systematic review and meta-analysis. Reprod Toxicol 2016;66:31-43. 10.1016/j.reprotox.2016.09.013. pmid:27667009. 27667009

[14] Thompson BL, Levitt P, Stanwood GD. Prenatal exposure to drugs: effects on brain development and implications for policy and education. Nat Rev Neurosci 2009;10:303-12. 10.1038/nrn2598. pmid:19277053. 19277053 PMC2777887

[15] Lauder JM, Wallace JA, Krebs H. Roles for serotonin in neuroembryogenesis. Adv Exp Med Biol 1981;133:477-506. 10.1007/978-1-4684-3860-4_28 pmid:7032250. 7032250

[16] Homberg JR, Schubert D, Gaspar P. New perspectives on the neurodevelopmental effects of SSRIs. Trends Pharmacol Sci 2010;31:60-5. 10.1016/j.tips.2009.11.003. pmid:19963284. 19963284

[17] Davies NM, Taylor G, Taylor AE, Martin RM, Munafò MR, Thomas KH. Cardiovascular and neuropsychiatric risks of varenicline: too good to be true?Lancet Respir Med 2015;3:e39-40. 10.1016/S2213-2600(15)00468-3 pmid:26679027. 26679027

[18] Lewis SJ, Relton C, Zammit S, Smith GD. Approaches for strengthening causal inference regarding prenatal risk factors for childhood behavioural and psychiatric disorders. J Child Psychol Psychiatry 2013;54:1095-108. 10.1111/jcpp.12127. pmid:24007416. 24007416

[19] Thomas KH, Martin RM, Davies NM, Metcalfe C, Windmeijer F, Gunnell D. Smoking cessation treatment and risk of depression, suicide, and self harm in the Clinical Practice Research Datalink: prospective cohort study. BMJ 2013;347:f5704 10.1136/bmj.f5704 pmid:24124105. 24124105 PMC3805476

[20] Idring S, Rai D, Dal H, et al. Autism spectrum disorders in the Stockholm Youth Cohort: design, prevalence and validity. PLoS One 2012;7:e41280 10.1371/journal.pone.0041280. pmid:22911770. 22911770 PMC3401114

[21] Idring S, Lundberg M, Sturm H, et al. Changes in prevalence of autism spectrum disorders in 2001-2011: findings from the Stockholm youth cohort. J Autism Dev Disord 2015;45:1766-73. 10.1007/s10803-014-2336-y. pmid:25475364. 25475364

[22] Källén B, Nilsson E, Olausson PO. Antidepressant use during pregnancy: comparison of data obtained from a prescription register and from antenatal care records. Eur J Clin Pharmacol 2011;67:839-45. 10.1007/s00228-011-1021-8. pmid:21387167. 21387167

[23] Stephansson O, Granath F, Svensson T, Haglund B, Ekbom A, Kieler H. Drug use during pregnancy in Sweden - assessed by the Prescribed Drug Register and the Medical Birth Register. Clin Epidemiol 2011;3:43-50. 10.2147/CLEP.S16305. pmid:21386973. 21386973 PMC3046184

[24] Peters L, Kapusnik-Uner JE, Nguyen T, Bodenreider O. An approximate matching method for clinical drug names. AMIA Annu Symp Proc 2011;2011:1117-26.pmid:22195172. 22195172 PMC3243188

[25] Ludvigsson JF, Andersson E, Ekbom A, et al. External review and validation of the Swedish national inpatient register. BMC Public Health 2011;11:450 10.1186/1471-2458-11-450. pmid:21658213. 21658213 PMC3142234

[26] Jörgensen L, Ahlbom A, Allebeck P, Dalman C. The Stockholm non-affective psychoses study (snaps): the importance of including out-patient data in incidence studies. Acta Psychiatr Scand 2010;121:389-92. 10.1111/j.1600-0447.2009.01500.x. pmid:19878139. 19878139

[27] Stuart EA, Rubin DB. Best Practices in Quasi-Experimental Designs: Matching methods for causal inference. In: Osborne J, ed. Best Practices in Quantitative Social Science.Sage Publications, 2007:155-76.

[28] Lee BK, Lessler J, Stuart EA. Improving propensity score weighting using machine learning. Stat Med 2010;29:337-46. 10.1002/sim.3782. pmid:19960510. 19960510 PMC2807890

[29] Frisell T, Öberg S, Kuja-Halkola R, Sjölander A. Sibling comparison designs: bias from non-shared confounders and measurement error. Epidemiology 2012;23:713-20. 10.1097/EDE.0b013e31825fa230. pmid:22781362. 22781362

[30] Smith GD. Negative control exposures in epidemiologic studies. Epidemiology 2012;23:350-1, author reply 351-2. 10.1097/EDE.0b013e318245912c. pmid:22317815. 22317815

[31] Lin DY, Psaty BM, Kronmal RA. Assessing the sensitivity of regression results to unmeasured confounders in observational studies. Biometrics 1998;54:948-63. 10.2307/2533848 pmid:9750244. 9750244

[32] Petersen I, Gilbert RE, Evans SJ, Man SL, Nazareth I. Pregnancy as a major determinant for discontinuation of antidepressants: an analysis of data from The Health Improvement Network. J Clin Psychiatry 2011;72:979-85. 10.4088/JCP.10m06090blu. pmid:21457681. 21457681

[33] Robinson EB, Samocha KE, Kosmicki JA, et al. Autism spectrum disorder severity reflects the average contribution of de novo and familial influences. Proc Natl Acad Sci U S A 2014;111:15161-5. 10.1073/pnas.1409204111. pmid:25288738. 25288738 PMC4210299

[34] Petersen I, McCrea RL, Lupattelli A, Nordeng H. Women’s perception of risks of adverse fetal pregnancy outcomes: a large-scale multinational survey. BMJ Open 2015;5:e007390 10.1136/bmjopen-2014-007390. pmid:26033946. PMC4458601 26033946

