# Supplementary material for: Antidepressants during pregnancy and autism in offspring: population based cohort study
Source: BMJ. 2017 Jul 19;358:j2811. doi: 10.1136/bmj.j2811 (PMC5516223; doi:10.1136/bmj.j2811)
Supplement: Supplementary file 1 — Appendix 1: Supplementary material [file raid037083.ww1.pdf]

Appendix: Methods supplement and supplementary tables [posted as supplied by author]

Methods Supplement

Propensity score matching

Propensity score matching was carried out on the sample used in Analysis 1. Children exposed to antidepressant use during pregnancy were matched with unexposed children who had mothers diagnosed with indications for antidepressant use before the child's birth (anxiety disorders, bipolar disorder, depression, non-affective psychoses, obsessive compulsive disorder, other stress related or neurotic disorders). This matching was performed based on the propensity score, the estimated probability of being exposed to antidepressants during pregnancy. Matching compares exposed and unexposed persons who are as similar as possible on the characteristics that determine exposure status. Importantly, this means that dissimilar individuals are removed from the analysis, thereby facilitating an 'apples to apples' comparison that is less likely to be confounded by the characteristics that predict exposure. An accessible discussion of propensity score matching may be found elsewhere. [1]

Propensity scores were estimated using a boosted classification and regression tree (CART) model. This machine learning algorithm has been shown to produce optimal estimates for maximizing bias reduction in propensity score adjustment. [2] Covariates in the model were: maternal age, sex, birth year, parity, maternal education, family income, maternal country of origin, maternal smoking at first antenatal visit, maternal body mass index at first antenatal visit, number and type of depression diagnoses (specialist care, primary care, inpatient care), and maternal neurologic and psychiatric conditions diagnosed before birth: (alcohol misuse, anxiety, bipolar disorder, obsessive-compulsive disorder, depression, drug misuse, non-affective psychotic disorders, other childhood disorders, other neurotic disorders, personality disorders, stress-related disorders, benign intercranial hypertension, bell's palsy, cerebrovascular disease, cerebral palsy, epilepsy, headache, migraine, myasthenia gravis, multiple sclerosis), and medications taken during pregnancy (anti-epileptics, anti-psychotics, and anxiolytics). Propensity score matching of exposed and unexposed individuals was carried out using a maximum of 4:1 unexposed:exposed nearest neighbour matching with a caliper of 0.20 SD and exact matching on birth year, sex of child, number of depression diagnoses, anxiety disorders, and obsessive-compulsive disorder. Matching was conducted using the *matchit* package in R. [3] Odds ratios and 95%CI are estimated from cluster robust logistic regression models.

The propensity score matched analyses for ASD, ASD without ID, and ASD with ID were based on the following sample sizes.

Outcome	N, total	N, matched exposed:unexposed	N, with outcome	N, exposed with outcome
ASD	6,426	1,608 : 4,818	177	63
ASD no ID	6,402	1,601 : 4,801	153	56
ASD with ID	6,273	1,552 : 4,721	24	7

Balance was assessed using the standardized mean distance, and overall matching success was assessed using the ASAM (average standardized absolute mean distance), where the closer to 0 the ASAM is, the better the covariate balance is. The ASAM before matching was 0.093. The ASAM after matching was 0.022. No individual covariate had a standardized difference greater than 0.10. A graphical display of the balance of each covariate in the overall matched sample of N = 6,426 is provided below.

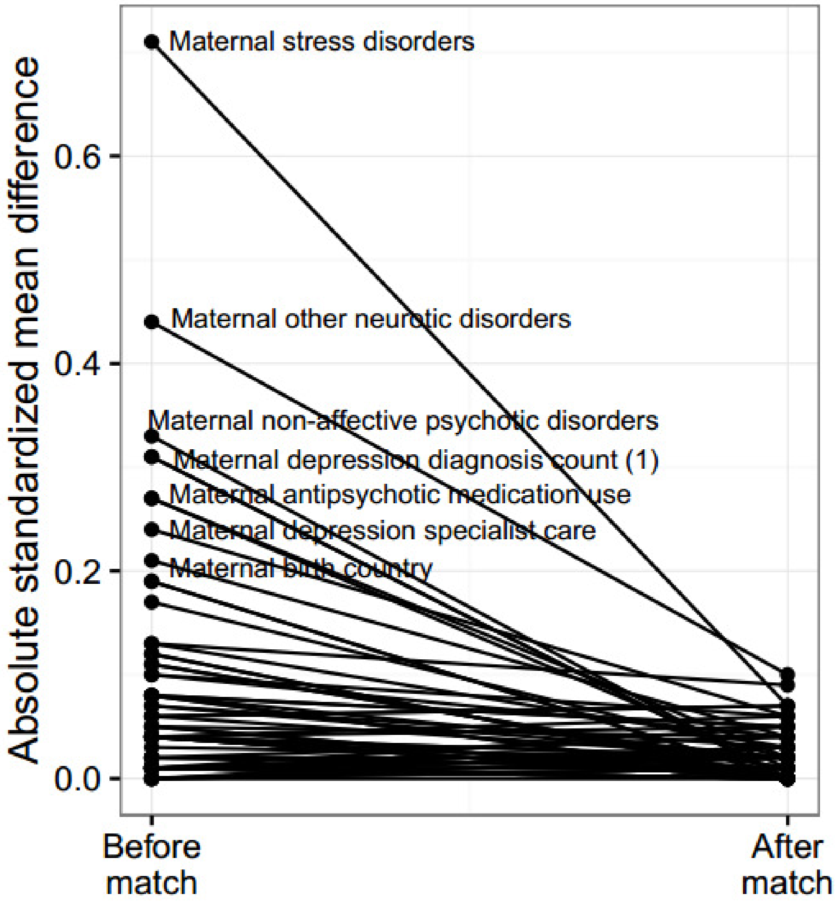

Sibling matching

Sibling matched analyses for ASD, ASD without ID, and ASD with ID were based on the following, respectively: 3,038 ASD cases, of whom 66 were exposed, and 94 sibling sets with discordant outcomes and discordant exposures; 2,408 ASD without ID cases, of whom 60 were exposed, and 81 sibling sets with discordant outcomes and discordant exposures; 630 ASD with ID cases, of whom 6 were exposed; 14 sibling sets with discordant outcomes and discordant exposures.

Missing data

In the study sample N = 254,610, there were missing data for the following characteristics: maternal age: N=5 (0.002%); paternal age: N=2284 (0.9%); family income quintile: N=117 (0.05%); maternal education: N=1395 (0.55%). These data were not imputed due to the small number missing. In Analysis 1, Model 3, missingness on these characteristics resulted in a sample loss of 1.5%. Missingness on these characteristics did not influence Analysis 2 because the propensity score estimation technique was able to incorporate the missingness in prediction. The sibling models in Analysis 3 did not adjust for these characteristics. In Analysis 4, missingness on these characteristics resulted in a sample loss of 1.0%. Therefore, missingness on these characteristics was not considered substantial.

Missingness was also observed for maternal smoking: N=34,249 (13.4%) and maternal BMI: N=52,893 (20.8%). Because smoking and BMI were shown to be not associated with ASD in our previous investigations in our study sample [4 5], these covariates were only included in the propensity score model. The boosted CART model as a non-parametric technique is able to incorporate missing values in prediction and as a result no sample loss due to missingness on smoking/BMI occurred in the propensity score analyses. Furthermore, since previously identified predictors of smoking/BMI missingness (e.g., birthyear) were also incorporated in the propensity score model; and because of the null associations of those characteristics with ASD, the missingness of these two characteristics is unlikely to meaningfully influence estimates.

References

1. Stuart EA. Matching methods for causal inference: A review and a look forward. Stat Sci 2010;25(1):1-21. doi: 10.1214/09-STS313 [published Online First: 2010/09/28]

2. Lee BK, Lessler J, Stuart EA. Improving propensity score weighting using machine learning. Statistics in medicine 2010;29(3):337-46.

3. MatchIt: MatchIt. R package version 2.3-1 [program], 2007.

4. Gardner RM, Lee BK, Magnusson C, et al. Maternal body mass index during early pregnancy, gestational weight gain, and risk of autism spectrum disorders: Results from a Swedish total population and discordant sibling study. Int J Epidemiol 2015;44(3):870-83. doi: 10.1093/ije/dyv081

5. Lee BK, Gardner RM, Dal H, et al. Brief report: maternal smoking during pregnancy and autism spectrum disorders. Journal of autism and developmental disorders 2012;42(9):2000-5. doi: 10.1007/s10803-011-1425-4 [published Online First: 2011/12/17]

SUPPLEMENTARY TABLES

Table A: Regression-estimated odds ratios and 95% CI for associations of antidepressant use during pregnancy and ASD when comparing children exposed prenatally to antidepressants ***prescribed for a known psychiatric condition*** with children exposed to maternal psychiatric disorders but no antidepressants.

**Outcome** (N exposed cases)	**Model 1**	**Model 2**	**Model 3**
ASD (75)	**1.72 (1.32 to 2.24)**	**1.53 (1.15 to 2.05)**	**1.48 (1.11 to 1.99)**
ASD without ID (66)	**1.80 (1.36 to 2.40)**	**1.65 (1.21 to 2.25)**	**1.57 (1.15 to 2.15)**
ASD with ID (9)	1.27 (0.63 to 2.53)	0.99 (0.44 to 2.21)	0.97 (0.41 to 2.28)

ID = intellectual disability
Cluster robust logistic regression models (cluster = birth mother)
Model 1: adjusted for birth year
Model 2: Model 1 + maternal psychiatric disorders diagnosed before birth (depression, anxiety disorder, bipolar disorder, non-affective psychotic disorders, obsessive-compulsive disorder, stress-related disorders, other neurotic disorders), maternal medications used during pregnancy (anti-epileptics, anti-psychotics, anxiolytics)
Model 3: Model 2 + sex, maternal age, paternal age, parity, maternal education, family income, maternal birth country

Table B: Sensitivity of model results to unobserved confounding at specified parameter levels.

Increase in risk of ASD on account of unmeasured confounder*	Prevalence of unmeasured confounder in exposed	Prevalence of unmeasured confounder in unexposed	Corrected OR (95% CI)
-	0	0	**1.76 (1.26 to 2.46)***
Doubled	10%	5%	1.68 (1.20 to 2.35)
Doubled	20%	10%	1.61 (1.15 to 2.25)
Doubled	30%	10%	1.49 (1.07 to 2.08)
Doubled	40%	10%	1.38 (0.99 to 1.93)
Doubled	50%	10%	1.29 (0.92 to 1.80)
Tripled	10%	5%	1.61 (1.15 to 2.25)
Tripled	20%	10%	1.51 (1.08 to 2.11)
Tripled	30%	10%	1.32 (0.95 to 1.85)
Tripled	40%	10%	1.17 (0.84 to 1.64)
Tripled	50%	10%	1.06 (0.76 to 1.48)

* assuming that the elevated risk of ASD due to *U* is consistent in both the exposed and the unexposed
** the original Table 3 propensity-score matched estimate of the OR and 95% CI for exposure to antidepressants during pregnancy and risk of ASD without intellectual disability

Odds ratios and 95% confidence intervals are calculated based on formulae provided by Lin et al., Biometrika 1998 http://www.jstor.org/stable/2533848

Table C: ASD rates by the most commonly prescribed antidepressants during pregnancy in the Stockholm Youth Cohort

Antidepressant	N exposed to drug	N, ASD cases (%)	N, ASD without ID (%)
Fluoxetine	453	16 (3.5%)	15 (3.3%)
Citalopram	1064	52 (4.9%)	46 (4.3%)
Paroxetine	264	5 (1.9%)	4 (1.5%)
Sertraline	912	31 (3.4%)	27 (3.0%)
Clomipramine	235	16 (6.8%)	15 (6.4%)
Venlafaxine	213	11 (5.2%)	11 (5.2%)
Any SSRI AD	2710	105 (3.9%)	93 (3.4%)
Any non-SSRI AD	723	32 (4.4%)	30 (4.1%)
High SERT affinity AD	1928	70 (3.6%)	63 (3.3%)
Low/moderate SERT affinity AD	1414	66 (4.7%)	59 (4.2%)

Note: Other antidepressants included were Fluvoxamine, Escitalopram, Amitryptiline, Mirtazapine, Bupropion and Duloxetine but results not presented since cell sizes for exposure or outcome group were <5.

AD = antidepressant; ID = intellectual disability; SERT = serotonin transporter

High SERT affinity antidepressants: escitalopram, fluoxetine, paroxetine, sertraline, duloxetine, clomipramine

Low/moderate SERT affinity antidepressants: citalopram, fluvoxamine, venlafaxine, amitriptyline, mirtazapine, bupropion

Table D: Odds ratios and 95% confidence intervals[1] for associations of the most commonly prescribed antidepressants during pregnancy and ASD in the Stockholm Youth Cohort

Antidepressant	ASD<br>OR (95% CI)	ASD without ID<br>OR (95% CI)
Fluoxetine	1.42 (0.84 to 2.39)	1.59 (0.92 to 2.73)
Citalopram	1.65 (1.20 to 2.26)	1.75 (1.25 to 2.45)
Paroxetine	0.61 (0.25 to 1.49)	0.60 (0.22 to 1.62)
Sertraline	1.45 (0.98 to 2.16)	1.52 (0.99 to 2.32)
Clomipramine	1.76 (1.01 to 3.05)	2.07 (1.17 to 3.64)
Venlafaxine	1.81 (0.89 to 3.71)	2.14 (1.05 to 4.37)
Any SSRI antidepressant	1.45 (1.14 to 1.83)	1.54 (1.20 to 1.97)
Any non-SSRI antidepressant	1.53 (1.03 to 2.28)	1.74 (1.15 to 2.63)
High SERT affinity AD	1.33 (1.02 to 1.73)	1.45 (1.09 to 1.92)
Low/moderate SERT affinity AD	1.71 (1.30 to 2.24)	1.84 (1.38 to 2.46)

AD = antidepressant; ID = intellectual disability; SERT = serotonin transporter

[1]OR and 95% CI compare persons exposed to the specific antidepressant against persons unexposed to any antidepressant but who have a prior maternal diagnosis of depression, anxiety disorder, bipolar disorder, non-affective psychoses, obsessive-compulsive disorder, stress-related disorders, or other neurotic disorders. Models are adjusted for birth year, maternal depression, and antidepressant polypharmacy (binary variable for use of 2 or more antidepressants).

High SERT affinity antidepressants: escitalopram, fluoxetine, paroxetine, sertraline, duloxetine, clomipramine; Low/moderate SERT affinity antidepressants: citalopram, fluvoxamine, venlafaxine, amitriptyline, mirtazapine, bupropion
